# What Domains of Belgian Euthanasia Practice are Governed and by Which Sources of Regulation: A Scoping Review

**DOI:** 10.1177/00302228231221839

**Published:** 2023-12-14

**Authors:** Madeleine Archer, Lindy Willmott, Kenneth Chambaere, Luc Deliens, Ben P. White

**Affiliations:** 1 Australian Centre for Health Law Research, 1969 Queensland University of Technology, Brisbane, QLD, Australia; 2 End-of-Life Care Research Group, 70493 Vrije Universiteit Brussel & Ghent University, Brussels, Belgium; 3 Department of Public Health and Primary Care, 26656 Ghent University, Ghent, Belgium

**Keywords:** assisted dying, euthanasia, regulation, scoping review, health law

## Abstract

Background: Multiple sources of regulation seek to shape euthanasia practice in Belgium, including legislation and training. This study comprehensively mapped which of these sources govern which domains of euthanasia practice, such health professionals’ obligations, or managing patient requests. Method: Scoping review methodology was used to search for scholarly records which discussed Belgian euthanasia regulation. Template analysis was used to generate themes describing the domains of euthanasia practice governed by sources of regulation. Results: Of 1364 records screened, 107 records were included. Multiple sources of regulation govern each domain, which are: the permissible scope of euthanasia; the legal status of a euthanasia death; the euthanasia process; the rights, obligations, and roles of those involved; system workings; and support for health professionals who provide euthanasia. Conclusions: Domains with significant yet fragmented regulation may lead to inconsistent care provision. Policymakers should develop coherent guidance to support health professionals to navigate this regulatory landscape.

## Introduction

Belgium was the third jurisdiction internationally after the US state of Oregon and the Netherlands to give legal protection to physicians who assist eligible individuals to die, in accordance with the requirements of the *Law on Euthanasia 2002* (the Act). In this article, ‘euthanasia’ means the intentional termination of life by a physician at the patient’s request. We recognise that internationally a range of terminology is used including ‘assisted dying’ and ‘medical assistance in dying’ (White & Willmott, 2021).

As with many international assisted dying systems, Belgian euthanasia practice is highly regulated. The Act governs which individuals may be eligible to access euthanasia and the process by which their eligibility is assessed (see Table 1 for a summary of the law). Provided all the legal requirements have been satisfied, terminally ill and non-terminally ill adults, terminally ill minors with the capacity to make decisions about euthanasia, and irreversibly unconscious adults who have made an advance directive on euthanasia may be eligible. The Act also establishes mechanisms for the monitoring and oversight of the system; all performed cases of euthanasia must be reported to the Federal Control and Evaluation Commission on Euthanasia (CFCEE) which reviews this information to ensure compliance with the Act.

**Table 1.** Summary of Legal Requirements in the Act (Archer et al., 2023).

	Requirements relating to the individual and their condition	Requirements relating to the process
Adults^a^ who are terminally ill	• Legally competent • Conscious when making the request • Experiencing persistent and unbearable and hopeless physical or psychological suffering that results from a serious and incurable illness caused by accident or disease • Expected to die within the foreseeable future • Physician, together with the patient, believes that there are no reasonable alternative solutions for the person’s suffering, that the patient is suffering physically or mentally constantly and unbearably, and is certain of the durable nature of the person’s request, taking into account the progress of the person’s condition	‘Standard procedural requirements’ • The request for euthanasia must be voluntary, considered and repeated, and not arrived at as a result of any external pressure • Written request • Physician’s duty to give information (diagnoses and prognosis, all therapeutic options, the assessment procedure) • Consultation with an independent physician
Adults^a^ who are not terminally ill	• As above, but *not* clearly expected to die within the foreseeable future	• Standard procedural requirements (above) • Second consultation with an independent psychiatrist or specialist in the person’s condition • One month waiting period between the person’s written request and the provision of euthanasia
Minors who are terminally ill	• Has ‘capacity for discernment’^b^ • In a medically futile state of constant and unbearable *physical* suffering that cannot be alleviated, and suffering is the result of a serious and incurable condition caused by illness or accident • Death is foreseeable • Conscious at the moment of making the request	• Standard procedural requirements (above) • Consultation with a child or adolescent psychiatrist or psychologist • Interview with the minor’s legal representatives, who give their written consent to the minor’s request
Adults^a^ who have made an advance directive requesting euthanasia	• Legally competent at the time the advance request is made • Irreversible state of unconsciousness according to the current state of medical science • Serious and incurable condition caused by illness or accident and condition is irreversible given the current state of medical science	• Advance directive drafted and signed as prescribed • Consultation with an independent physician • Nursing team and adult confidant^c^ are consulted about the advance directive • The advance directive is valid for an indefinite period

^a^Under the Act, minors with legal capacity (or ‘emancipated minors’) are treated as adults. These are mature minors who, through marriage or a court decision have legal capacity to make their own decisions.

^b^The concept of ‘capacity for discernment’ is similar to that of ‘Gillick competence’ used to refer to mature minors who are able to understand the consequences of their request for euthanasia.

^c^Under the Act, an adult confidant is a person who informs the person’s attending physician about the person’s advance directive requesting euthanasia. They may be appointed by the person drawing up the advance directive. More than one confidant may be appointed, but the person’s attending physician, the independent consulted physician, and members of the person’s nursing team are precluded from acting as confidants.

Physicians who provide euthanasia must comply with the Act; the law therefore is said to ‘regulate’ their conduct. However, law is not the only source which guides or influences euthanasia practice in Belgium. In this article, we collectively refer to these sources which seek to guide behaviour as ‘sources of regulation.’ They include law, policies, professional standards, and training programs. In the Belgian context, most studies to date have focused on a single source of regulation. For example, some literature examines institutional policies on euthanasia (D’Haene et al., 2009; Lemiengre et al., 2007, 2010), a community-initiated end-of-life training program called Life End Information Forum (LEIF) (Van Wesemael et al., 2009, 2010; Vissers et al., 2022), and guidelines for assessing patients whose request for euthanasia is based on mental illness (Verhofstadt et al., 2019, 2020, 2022).

Recently, attention has been given to a holistic view of regulation, and comprehensively mapping sources of regulation, rather than siloing these discussions and considering one source of regulation in isolation from others (White et al., 2022). Seeking to advance this new understanding, a recent scoping review (mapping review) mapped the sources of regulation operating in the Belgian euthanasia system (Archer et al., 2023). It identified that six broad sources of regulation seek to shape euthanasia practice: law, policy, professional standards, training programs, advisory documents, and system infrastructure (Table 2).

**Table 2.** Description of Each Source of Regulation Identified as Governing Belgian Euthanasia Practice (Archer et al., 2023).

Law
Refers to legal instruments which state the law on euthanasia, including the Act, royal decrees, and case law. As such, the term ‘law’ does not only refer to the Act on Euthanasia but is a broader term which encompasses other forms of law which explicitly govern euthanasia.
Policy
Describes written policies on the subject of, or relating to, euthanasia produced by government departments, and health care organisations and institutions. These bodies are directly involved in regulating euthanasia (government departments) or are health care organisations which might be expected to provide euthanasia (whether they permit this practice or not). These documents do not have the binding force of the law.
Professional standards
Refers both to written documents and disciplinary proceedings concerning euthanasia practice authored or initiated by professional (including medical, nursing, and pharmaceutical) associations and organisations.
Training programs
Describes educational programs which address euthanasia as a component of the curriculum, including tertiary (undergraduate, post-graduate) education and non-mandatory community-initiated regional training programs.
Advisory documents
Refers to documents about euthanasia produced by individuals or organisations which do not provide euthanasia. These documents provide clarifications and interpretations of aspects of euthanasia practice and are advisory in nature.
System infrastructure (or system design)
Covers top-down (government-lead) and bottom-up (community-initiated) infrastructure which performs a structural role in the regulatory framework and supports the implementation of the law into practice. Includes both instruments (written documents, forms, or databases) and organisations/entities which play a role supporting the system’s operation. Includes infrastructure created by and pursuant to the Act, as well as infrastructure created after, or independently of the Act, such as community-initiated services without statutory authority or government mandate.

While that study was useful for mapping all the sources of regulation operating in this system and advancing a holistic view of euthanasia regulation in Belgium, it did not identify the domains of euthanasia practice that those sources seek to govern. In other words, it did not identify whether sources of regulation provide guidance on, for example, how medical practitioners might assess a person’s eligibility for euthanasia, or how the mandatory consultation of an independent physician should occur.

Consistent with regulatory scholarship, ‘domains’ is understood in this article as the ‘areas of social life that are being regulated’ or the ‘range of issues’ about which regulatory decisions are made (Black, 2002; Hancher & Moran, 1989). To illustrate, a hospital’s policy on euthanasia might govern caregivers’ roles in providing euthanasia. The public prosecutor may govern what acts might or might not constitute euthanasia. In these examples, caregivers’ roles and the acts that constitute euthanasia are the domains of euthanasia practice being governed by the policy and the public prosecutor, respectively.

Significantly, the domains of euthanasia practice governed by regulation are generally understudied. Some studies have provided insight into some domains, for example, interpreting the eligibility (Schweitser et al., 2021; Van Assche et al., 2019) and independent consultation requirements (Cohen et al., 2014; Van Wesemael et al., 2010). However, not all domains of euthanasia practice governed by all sources of regulation have been comprehensively identified. Undertaking this exercise is important. It may identify that certain domains of practice are heavily governed by several different sources of regulation, meaning that providers have numerous guidelines to be aware of, navigate, and reconcile in their practice. Existing research has found some variation in how euthanasia is provided between health care providers and across regions (Cohen et al., 2012; Lemiengre et al., 2008; Verhofstadt, Audenaert, Van den Broeck, Deliens, Mortier, Titeca, Pardon, De Bacquer, et al., 2020) including health care providers adopting protocols for euthanasia which are more stringent than the legal requirements (Gastmans, Lemiengre, & de Casterlé, 2006; Lemiengre et al., 2008; Verhofstadt, Audenaert, et al., 2019; Verhofstadt et al., 2019). Inconsistencies in euthanasia implementation may lead to patients being treated differently across health care settings, or unfairly excluded from access to a legal end-of-life option. In addition, imposing different constraints on health professionals’ practice may cause frustration or confusion, and inhibit their ability to assist their patients.

This study sought to address this gap, through drawing on existing literature, to identify (a) the broad domains of euthanasia practice governed by sources of regulation in Belgium, and (b) which sources of regulation govern which domains of practice.

## Methods

Scoping reviews are useful for mapping and summarising literature in a field of study (Arksey & O’Malley, 2005). This methodology permitted us to comprehensively review published scholarly literature to collect and integrate information on the domains of euthanasia practice governed by the sources of regulation that were identified by the mapping review (Archer et al., 2023), namely, law, policy, professional standards, training programs, advisory documents, and system infrastructure (Table 2).

The review was guided by Arksey and O’Malley’s five-stage methodological framework for scoping studies: identifying the research question(s), identifying relevant studies, selecting relevant studies, charting the data, and collating, summarising, and reporting the results.

### Identifying the Research Questions

The two research questions in this study were as follows. First, what are the domains of euthanasia practice that the literature identifies as being governed by Belgian euthanasia regulation? Second, which sources of regulation govern which domains of euthanasia practice? As can be seen from Table 2, some sources of regulation identified are instruments (published, written materials) and others are organisations (e.g., the public prosecutor).

### Identifying Relevant Studies and Study Selection

The scoping review protocol developed in the mapping review (Archer et al., 2023) was used and tailored for this study (Supplementary File 1). Searching for book chapters and journal articles in peer reviewed journals was undertaken in six interdisciplinary databases and in reference lists of included records. An initial search of the databases was conducted on 11/2/22. Two further database searches were undertaken on 9/3/22 and 25/3/22 which expanded the search to include a broader range of records.

Records’ eligibility for inclusion in the study was screened by MA and BPW through title, abstract, and full-text review, and were included where they met formal and substantive inclusion criteria (Table 3). This process identified a number of records which met the inclusion criteria, and were subject to data extraction and analysis (described below).

**Table 3.** Inclusion Criteria Applied in the Scoping Review (Archer et al., 2023).

Formal inclusion criteria	Book chapter or journal article in a peer reviewed^a^ journal
	Full text available
	Published 28 May 2002 (inclusive) to 2022 (the date on which the relevant search was conducted)^b^
	Written in English, Dutch, or French
Substantive inclusion criteria	Belgian regulation of euthanasia is a substantive focus of the record
	Record has substantive discussion, analysis, or engagement with one or more specific sources of regulation operating in the present Belgian euthanasia legal framework

^a^A journal was considered to be peer reviewed unless web searching suggested that it was not. Ulrichsweb Global Serials Directory was used to facilitate this process but the absence of a ‘refereed’ icon was non-determinative of a lack of peer review and further searching was undertaken.

^b^The end-dates for each of the three searches were 11/02/22, 09/03/22 and 25/03/22.

The complete method for data collection (including search strategy information) is reported in Supplementary File 2 given it has already been reported elsewhere (Archer et al., 2023). This study is reported consistent with PRISMA-ScR guidelines (Tricco et al., 2018), noting that some items are reported in Supplementary Materials.

### Charting the Data

First, all records were read in full without annotation to ensure data familiarisation. DeepL, an online translation program, was used to translate Dutch and French texts into English. DeepL produced high-quality translations in which regulatory sources and discussions about them were easily identified and understood. Records were read a second time, and any discussion about each source of regulation (law, policy, professional standards, training programs, advisory documents, and system infrastructure: Table 2) were highlighted. The domains of euthanasia practice that each source of regulation was described as governing were also highlighted.

Second, a coding chart or ‘template’ was developed in Microsoft Excel to extract and organise the highlighted data. The identified sources of regulation were listed in the template (see Table 4 for an overview of these, including the sub-types for each source of regulation and examples of each). Then, data from each record about the domains of euthanasia practice that each source of regulation was described as governing was also entered into the template, verbatim, or in their truncated form. To illustrate, one record discussed the domains of practice governed by the LEIF modules as being: end-of-life decisions, the actual practice of euthanasia, and communicating about euthanasia. In this instance, data about ‘end-of-life decisions,’ ‘euthanasia practice,’ and ‘communicating about euthanasia’ were extracted into the template in relation to the ‘training programs’ source of regulation (Van Wesemael, Cohen, Onwuteaka-Philipsen, Bilsen, Distelmans, et al., 2009).

**Table 4.** Overview of the Sources of Regulation, Indicating Source Sub-types and Giving Examples for Each (Archer et al., 2023).

Source of regulation and sub-types	Example
Law	
Legislation, amendments to the law	*The Act*
Royal decrees	*Royal Decree of 2 Apr 2003* establishing the procedures for drawing up, reconfirming, revising or withdrawing the advance declaration on euthanasia
Case law	Constitutional court judgement 14 Jan 2004 – Judgement number 4/2004
Policy	
Organisation-level policies	Caritas Flanders policy: Caring for a dignified End of Life (Zorg voor een menswaardig levenseinde), 2005
Institution-level policies	Ghent University procedure concerning euthanasia and psychological suffering, 2009
Public policy	Federal Health department Circular addressed to physicians, “Advance requests for euthanasia – electronic consultation by physicians” 4 Sep 2008
Professional standards	
Written standards (medical, psychiatric, general practice, pharmaceutical)	Order of physicians Advice of Mar 2003 regarding palliative care, euthanasia, and other medical decisions at the end of life
Disciplinary proceedings	Decision 24 Oct 2007 Provincial Council of West Flanders (Order of Physicians)
Training programs	
Tertiary education	Vrije Universiteit Brussel undergraduate core curriculum
Non-mandatory training	Life End Information Forum (LEIF) training on euthanasia
Advisory documents	
Produced by independent statutory bodies (the CFCEE and the National Bioethics Committee)	CFCEE biannual reports on euthanasia practice
Academic articles	Beatrice Figa, ‘L’euthanasie: Considérations <practiques>’ [2006] 230 La Revue de la Médecine Générale 82
System infrastructure	
Pre-existing system infrastructure	Office of the public prosecutor
Created pursuant to the Act	CFCEE registration document
Developed independently of the Act	End-of-life consultation and advice centres (LEIF, Forum End of Life, ULTeam)

Descriptive information about each record was extracted into a separate Microsoft Excel spreadsheet, including the record’s citation, language, and a summary of its primary content.

### Collating, Summarising, and Reporting the Results

The charted data was thematically analysed by MA using ‘template analysis,’ a ‘codebook’ approach to thematic analysis (Braun & Clarke, 2006; King, 2004). The template used to extract and organise the data functioned as a structured platform for the inductive generation of semantic themes (Braun & Clarke, 2022). First, MA sorted the data to produce specific, descriptive codes. Next, these codes were re-organised and adapted to produce overarching themes which described the broad domains of euthanasia practice governed by the sources of regulation. As the analytical process occurred within the template, each source of regulation was mapped to the domains of practice it governed. The final themes/domains and sub-domains were discussed and settled by all authors.

The descriptive information obtained from each record was analysed to produce descriptive statistics for the records included in the final review.

## Results

### Record Characteristics

**Figure 1.**
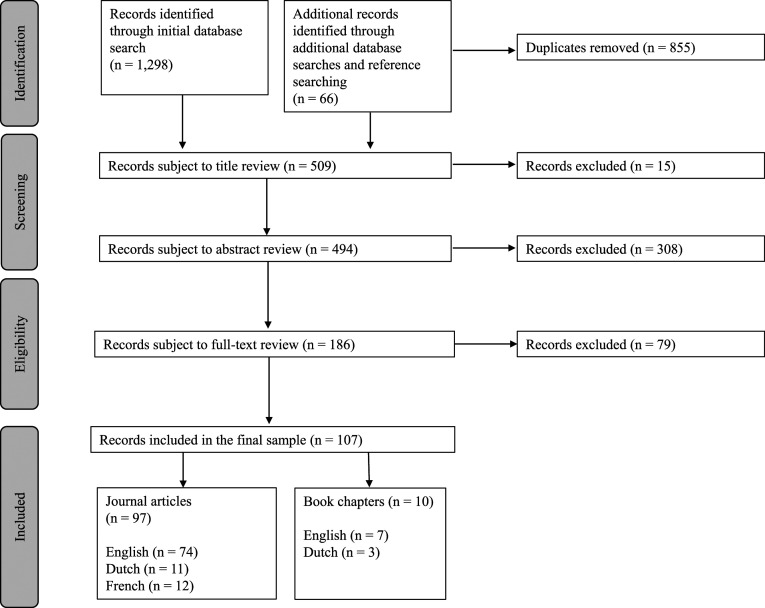
Diagram reflecting the outcomes of the screening and eligibility review processes applied by Archer et al. (2023).

A total of 107 records were included in the review, from an initial 1364 records generated from the database and reference list searches (Figure 1). The reason for exclusion at each stage of the review process was non-adherence to the formal or substantive inclusion criteria. For illustration, records were excluded where their primary focus was to critique other scholarly work, or where they sought to apply a specific philosophical, rights-based, or theoretical lens to euthanasia practice.

The records included in the review were mostly journal articles (*n* = 97), records written in English (*n* = 81) and records reporting non-empirical studies (*n* = 74). Fewer records were book chapters (*n* = 10), written in Dutch (*n* = 14) or French (*n* = 12), and those reporting empirical research (*n* = 33).

Supplementary File 3 contains the descriptive information extracted from each record.

### Themes on the Governed Domains of Euthanasia Practice

Template analysis identified six themes which reflect the domains of euthanasia practice described in the included records as being governed by sources of regulation. These are: the permissible scope of euthanasia; the legal status of a euthanasia death; the euthanasia process; rights, obligations, and roles of those involved; system operation; and support for health professionals who provide euthanasia care. Sub-domains were generated for each practice domain. A table depicting the domains of practice governed by each source of regulation was also produced (Table 5).

**Table 5.** Depiction of the Themes Generated in the Analysis (the Domains and Sub-domains of Euthanasia Practice Governed by Sources of Regulation in Belgium) Which Maps Each Source of Regulation to Each Domain of Practice.

			Source of regulation					
			Law	Policy	Professional standards	Training programs	Advisory documents	System infrastructure
Domain and sub-domains of euthanasia practice governed	The permissible scope euthanasia	Defining euthanasia	●		●	●	●	●
		Defining who is eligible for euthanasia	●	●	●			
	The legal status of euthanasia death	-	●		●			
	The euthanasia process	Identifying and managing a patient’s request for euthanasia^a^	●	●		●	●	●
		Assessing a person’s eligibility	●	●	●	●	●	●
		Navigating consultation requirements	●	●	●	●	●	●
		The role of palliative care in the euthanasia process		●	●	●		
		Administering the life-ending medication	●	●	●	●	●	
		Duration of the euthanasia process for patients whose request is based on mental illness	●	●	●		●	
		Post-death processes		●	●			
	Rights, obligations, and roles of those involved	Rights of providers and patients	●	●	●		●	●
		Health professionals’ obligations	●	●	●		●	●
		Roles of providers, patients, and their relatives	●	●	●	●	●	●
	System operation	Facilitating supply and/or resourcing of the euthanasia substances	●		●			
		Facilitating the CFCEE’s functions	●					●
		Facilitating independent consultations						●
	Support for health professionals who provide euthanasia care	Providing ethical, professional, high quality care		●	●	●	●	●
		Need for provider support		●	●	●		

^a^While framed broadly in terms of requests for euthanasia, the governance which falls under this sub-domain of euthanasia practice relates mostly to identifying and managing a person’s *advance* request for euthanasia, that is, a *non-contemporaneous* request for euthanasia.

#### Practice Domain 1: The Permissible Scope of Euthanasia

Several sources of regulation define the permissible scope of euthanasia. Two sub-domains were generated in relation to this domain: defining euthanasia under the legal framework and defining who is eligible for euthanasia.

##### Defining Euthanasia

Law, professional standards, training, advisory documents, and system infrastructure govern how euthanasia is defined pursuant to the legal framework. By prescribing that euthanasia is intentionally terminating life by someone other than the person concerned, at the latter’s request, the Act is understood to legalise ‘voluntary euthanasia’ performed by a physician (Mroz et al., 2021).

However, sources of regulation other than law provide guidance on whether ‘physician assisted suicide’ (PAS), where the medication is taken by the patient, falls within the scope of the law. Advice issued by the national medical association (professional standards) (Nationale Raad Van De Orde Der Geneesheren, 2004), and materials produced by the CFCEE (advisory documents) (CFCEE, 2005) explain and distinguish euthanasia and PAS. Both advise that if all the requirements of the Act are followed and the physician remains present to oversee the performance, PAS falls under the Act, and is therefore permitted. This is because the Act itself does not prescribe the relevant administration method.

Disciplinary and criminal proceedings (professional standards and law) and the public prosecutor (system infrastructure) govern how euthanasia is defined by applying the statutory definition to factual scenarios, for example, by determining whether a health practitioner administering morphine to a patient in the relevant circumstances was ‘euthanasia’ as defined in the legal framework.

Finally, training programs distinguish euthanasia from other forms of end-of-life care, and other legal frameworks. For example, the LEIF training situates euthanasia in the context of end-of-life care and legal frameworks on palliative care and patient rights (Van Wesemael, Cohen, Onwuteaka-Philipsen, Bilsen, Distelmans, et al., 2009). Other training programs consider how euthanasia fits into conventional palliative care, managing pain at the end of life, palliative care problems, and the practical implications of euthanasia.

##### Defining who is Eligible for Euthanasia

Law, policies, and professional standards define who is eligible for euthanasia. The Act contains broad statements of eligibility, including medically hopeless situation, and persistent and unbearable suffering. The 2014 amendment to the law, and a constitutional court judgement extend those eligible to minors with the capacity for discernment.

Policies define the permissibility of euthanasia within their institution or organisation, by accepting the eligibility conditions established in the law, or narrowing the types of patients who might otherwise be eligible. For example, Caritas Catholica, a health service umbrella organisation, provides that patients must be conscious, of age (namely over 18), and have a terminal illness to access euthanasia in their institutions. This definition functionally excludes many patients who would otherwise be eligible under the Act.

Professional standards provide that euthanasia is appropriate and permissible when the Act is complied with. Guidelines for psychiatry accept euthanasia for patients whose request is based on mental illness, though they advise that psychiatrists apply particular care and caution in this area and with these patients (Lemmens, 2018; Vlaamse Vereniging voor Psychiatrie, 2017).

#### Practice Domain 2: Legal Status of Euthanasia Death

Both law and professional standards provide advice on the legal status of a euthanasia death. The Act prescribes that euthanasia is not suicide for the purposes of insurance and other contracts. The Order of Physicians’ 2003 advice (professional standards) provides that physicians should not indicate euthanasia as the cause of a person’s death on the death certificate for the purposes of insurance (Balthazar, 2003).

#### Practice Domain 3: The Euthanasia Process

All sources of regulation govern the different parts of the euthanasia process. Seven practice sub-domains were generated under this practice domain: identifying and managing a patient’s request for euthanasia; assessing a person’s eligibility for euthanasia; navigating consultation requirements; the role of palliative care in the euthanasia process; administering the life-ending medication; the duration of the euthanasia process for patients whose request is based on mental illness; and post-death processes.

##### Identifying and Managing a Patient’s Request for Euthanasia

Law, policy, training programs, advisory documents, and system infrastructure govern the identification and management of a euthanasia request. Some guidance relates to managing a contemporaneous request for euthanasia. For example, one policy directs caregivers to be willing to listen to the person’s request and ask a number of probing questions to clarify the request (Gastmans et al., 2004).

Most of the regulation discussed in this sub-domain considers the possibility and potential validity of advance directives requesting euthanasia. It also provides guidance on identifying legal advance directives on euthanasia, and processes for their drafting, registration, confirmation, withdrawal, and access. The national database for the registration of advance directives (system infrastructure) facilitates the creation and registration of these documents.

##### Assessing Eligibility

All sources of regulation provide guidance on assessing a person’s eligibility for euthanasia.

Law, policies, professional standards, training, and advisory documents all govern how patients’ eligibility for euthanasia is assessed. While the Act largely defines who is eligible for euthanasia, constitutional case law, for example, also provides specific information on how individuals’ eligibility should be assessed. For example, a 2015 decision of the court provided advice on assessing a minor patient’s ‘capacity for discernment’ (Veny, 2015).

There are several eligibility criteria that sources of regulation other than the law commonly provide guidance on. These include: the incurability and the non-alleviable nature of the patient’s condition; the patient’s unbearable (physical or mental) suffering; the foreseeability of the patient’s death; and the voluntariness and well-considered nature of the patient’s request. For example, the Flemish Psychiatric Association (VVP) guideline (a professional standard) directs that for a condition to be incurable, all indicated medical treatments must have been implemented rather than merely considered. Where a patient refuses treatment, this is their right, but will likely mean that their condition cannot be considered incurable (Verhofstadt, Audenaert, et al., 2019).

Some sources of regulation also provide guidance on assessing patients with particular conditions, for example, patients with poly-pathology, those who are tired of life, and those who have neurocognitive disorders or mental illness. For example, the ninth report of the CFCEE (an advisory document), advises that a person might be eligible for euthanasia based on poly-pathology, or ‘multiple disorders’ such as reduced eyesight, early stage dementia, and incontinence (Raus et al., 2021).

##### Navigating Consultation Requirements

Under the Act, an independent physician must be consulted to provide an advice on the person’s eligibility (see Table 1). All sources of regulation provide guidance on navigating this process. Non-legal sources of regulation define ‘independence,’ operationalise the consultant’s qualifications and expertise, and define the roles of each physician in undertaking this exercise. For example, the CFCEE’s first biannual report (an advisory document) defines independence as no family or hierarchical tie between the physicians, and no therapeutic relationship with the patient. The consultation service developed by LEIF (system infrastructure) is specifically intended to facilitate this independent consultation.

The Act is silent on the effect that the consultant’s advice has on the person’s eligibility, but other sources specifically consider this issue. For example, the VVP guideline (a professional standard) goes much further than the law in directing that euthanasia is only possible for patients whose request is based on mental illness after *two uniformly positive opinions* from at least *two consulted psychiatrists* (Verhofstadt, Audenaert, et al., 2019). A 2015 judgement of the constitutional court has provided clarification on this issue for minors with the capacity for discernment: both independent consultations required must be positive to progress the request. That judgement also clarified the definition of ‘independence’ in the case of these patients (Constitutional Court, 2015).

##### The Role of Palliative Care in the Euthanasia Process

Policies, professional standards, and training programs govern the role of palliative care in the euthanasia process. Policies and professional standards emphasise that palliative care should feature in a patient’s euthanasia assessment trajectory. For example, some guidelines require that patients who make a request for euthanasia undergo consultation with a palliative care team before their request may be progressed (Gastmans, 2002). Training programs more generally consider the relationship between palliative care, euthanasia, and end-of-life care, pain management, and the legal framework on palliative care.

##### Administering the Life-Ending Medication

Law, policy, professional standards, training programs, and advisory documents govern the euthanasia administration process. Collectively, these sources of regulation provide guidance on accessing and obtaining the medication from the pharmacist; pharmacists’ duties and roles in facilitating prescriptions and the delivery of the medication; what medications to use and in what order; where to administer (in terms of the patient’s external environment); which practitioner should administer; preparing for death; medication protocols; return of unused medication; ordering and pricing of medications; and clinical aspects of administration. To illustrate, the Walloon Forum End of Life training addresses clinical facets of the administration process including identifying an access route (Lossignol, 2014) and pharmaceutical professional guidelines provide in-depth guidance on the delivery and return of the lethal medications (Meek, 2006).

##### Duration of the Euthanasia Process for Patients Whose Request is Based on Mental Illness

Laws, policies, professional standards, and advisory documents govern the duration of the euthanasia process from the time of the person’s request to the performance of euthanasia. This advice relates only to patients requesting euthanasia on the basis of mental illness. The Act instils a minimum one-month waiting period for these patients between their request for euthanasia and its performance. Guidelines vary regarding whether they adopt this one-month waiting period in the Act, or whether they choose to extend it to six months, or 12 months (Verhofstadt et al., 2019, 2020).

##### Post-Death Processes

Policy and professional standards govern the process once the person has received euthanasia. The Ghent University Hospital’s 2010 procedure on euthanasia and psychological suffering (policy) mandates that the person’s death certificate is to be completed only by the attending psychiatrist (Verhofstadt, Audenaert, et al., 2019). The Order of Physicians’ 2003 advice (a professional standard) provides instruction on the appropriate completion of the death certificate (Balthazar, 2003). Only institutional policies govern aftercare for the patient’s relatives and caregivers, which is largely undertaken by nurses (Lemiengre et al., 2007).

#### Practice Domain 4: Rights, Obligations, and Roles of Those Involved

Several sources of regulation govern this practice domain, which is divided into three sub-domains: rights of providers and patients; health professionals’ obligations; and the roles of providers, patients, and their relatives.

While the concepts of rights, obligations, and roles are distinct, they are discussed together in this practice domain because they intersect in many respects. For example, a physician’s *role* in euthanasia is contingent upon the exercise of their *right* to choose whether to participate in the practice. Regardless of the exercise of this right, when a patient makes a request for euthanasia to a physician, they have *obligations* to the patient, either in respect of conducting an assessment or referring the patient to a participating practitioner or service.

##### Rights of Providers and Patients

Law, policies, professional standards, advisory documents, and system infrastructure govern providers’ and patients’ rights in relation to euthanasia.

In terms of the rights of *providers*, sources of regulation observe or clarify these rights. Some sources of regulation provide guidance on physicians’ ability to be remunerated for their euthanasia work, and reiterate individuals’ and institutions’ ability to refuse to participate in or provide euthanasia. Sources of regulation also emphasise physicians’ rights to add conditions upon patient access to euthanasia in addition to those set out in the Act, for example, to impose a ‘palliative filter’ upon patient access. Pharmacists’ rights are not governed uniformly. While pharmacists have a general right to conscientiously object to euthanasia, one record describes that a guideline produced by the Order of Pharmacists (a professional standard) prevents pharmacists from objecting to dispensing medication used for euthanasia (Lossignol, 2014). Some sources of regulation attempt to curtail providers’ rights in respect of euthanasia. Records referred to a publicly available report of the National Bioethics Committee (an advisory document) as dis-endorsing healthcare institutions’ perceived right to invoke a collective conscientious objection clause to justify the non-provision of euthanasia onsite (Lossignol, 2016).

In terms of *patients’* rights, sources of regulation emphasise patients’ rights to request (but not receive) euthanasia, to give or withhold consent to treatment, and to request that an objecting physician transfers their medical file to a new practitioner willing to assist them with their request for euthanasia. Consultation and advice centres on euthanasia (system infrastructure) facilitate patients’ rights to ask for euthanasia.

##### Health Professionals’ Obligations

Law, policy, professional standards, advisory documents, and system infrastructure govern health professionals’ obligations in relation to euthanasia practice. A primary obligation imposed by the Act requires physicians who have performed euthanasia to report this to the national oversight body, the CFCEE. In some institutions, the physician might also be required by local policy to report the euthanasia to internal or institutional bodies. An amendment to the Act passed in 2020 imposes a referral obligation on physicians where a patient makes a request to them and they refuse/conscientiously object. These physicians are now required to refer the person to a euthanasia organisation. The Act also refers to an obligation of the care team; they are not permitted to benefit from a patient’s will.

The public prosecutor (system infrastructure), through its role to enforce compliance with the Act, reinforces the obligation on physicians to report the performance of euthanasia.

##### Roles of Providers, Patients, and Their Relatives

All sources of regulation govern one or more of the roles of providers, patients, and their relatives in euthanasia practice. Sources of regulation delineate caregiver roles, prescribe how care teams should interact, and how patients and relatives should be involved in decision-making. For example, one record reports a content analysis which revealed that Flemish nursing home policies delineate the role of GPs expansively; their roles include listening to the resident’s request for euthanasia, informing the resident about their options, coordinating the resident’s euthanasia trajectory, performing the euthanasia, and completing the relevant paperwork once the resident has died. This study also spoke to the role of the patient and their relatives as described in these policies. Patients’ roles are largely passive: to be informed in relation to a number of matters, and to receive care in a variety of forms. Some active roles that patients might have in the process include deciding who they want to know about their request for euthanasia, engaging in decision-making about their death, and working with the care team on a ‘euthanasia care plan’ (Lemiengre et al., 2009).

#### Practice Domain 5: System Operation

Law, professional standards, and system infrastructure govern how the euthanasia system operates. Three practice sub-domains were generated in relation to this practice domain: facilitating supply and/or resourcing of the euthanasia substances; facilitating the CFCEE’s functions; and facilitating independent consultations.

##### Facilitating Supply and/or Resourcing of the Euthanasia Substances

Pharmaceutical professional standards and law, via an amendment in 2005, govern pharmacological supply to ensure that the system is pharmaceutically viable.

##### Facilitating the CFCEE’s Functions

The Act and royal decrees (law), and the CFCEE registration document (system infrastructure) facilitate the CFCEE’s functions. These sources govern how the CFCEE undertakes its monitoring and oversight functions, and how its members are appointed.

##### Facilitating Independent Consultations

End-of-life consultation and advice centres (system infrastructure) govern system operation by facilitating the independent consultation required by the Act. These centres provide services which connect an attending physician with a physician who may be able to conduct this assessment.

#### Practice Domain 6: Support for Health Professionals who Provide Euthanasia Care

All sources of regulation other than law govern the provision of support to health professionals who provide euthanasia care and/or engage in decision-making about euthanasia for patients who request it. The two practice sub-domains generated in relation to this practice domain were providing ethical, professional, high-quality care, and the need for provider support.

##### Providing Ethical, Professional, High-Quality Care

All sources of regulation except law govern the provision of ethical, professional, high-quality care. This sub-domain intersects with practice domain 3, as some of the governance in this sub-domain is process related.

Organisational policies and training programs emphasise the need for decision-making to reflect ethical values.

Institutional policies, professional standards for physicians and psychiatrists, and advisory documents emphasise providing care that is professional, high-quality, or ‘best practice.’ In this vein, sources of regulation set out specific communication techniques such as listening to the request, providing information, and asking probing questions, encourage transparency in discussions with the patient and specialised, patient-centred care and counselling (which considers the spiritual, social, and psychological aspects of the euthanasia request). Consultation and advice centres also govern this sub-domain by providing care for patients who were assessed as ineligible. Emphasising professionalism, professional standards highlight the need for timeliness, open communication, interdisciplinary communication and information-sharing. In operationalising ‘best practice’ in psychiatry, the VVP guideline recommends that the care and assessment of patients with psychiatric disorders is comprised of a life and a death track, which ensures their options both for living and dying are fully explored. The guideline also advises documentation, having face-to-face discussions, and taking measures to maintain a trusting relationship with the patient (Verhofstadt, Audenaert, et al., 2019).

##### Need for Provider Support

Policies, professional standards, and training programs identify the need for euthanasia providers to receive support, and then providing mechanisms for such support. For example, the LEIF training program recognises the emotional and psychological impact of euthanasia practice on physicians, and the support it provides to physicians is practical, as well as technical and emotional. One record analysed the content of Flemish hospital policies on euthanasia (Lemiengre et al., 2008). The authors describe some of these policies as providing for caregivers to receive psychological or spiritual support. They set out a procedure for health professionals with a conscientious objection to euthanasia to access a point-of-contact to seek advice in respect of their concerns, and identify avenues and processes by which conflicts between caregivers in the euthanasia decision-making process might be resolved.

## Discussion

### Summary of Main Findings

We identified six themes which describe the governed domains of euthanasia practice in Belgium: the permissible scope of euthanasia; the legal status of a euthanasia death; the euthanasia process; the rights, obligations, and roles of those involved; system operation; and support for health professionals who provide euthanasia care. These domains incorporate 17 sub-domains.

We also mapped each source of regulation, consisting of law, policy, professional standards, training programs, advisory documents, and system infrastructure, to the domains of euthanasia practice that they govern. Each domain is governed by multiple, different sources of regulation, though some are more heavily governed than others. For example, the permissible scope of euthanasia, the euthanasia process, and the rights, obligations, and roles of those involved, are governed by all six sources of regulation. Within these practice domains, the most heavily governed practice sub-domains are assessing a person’s eligibility for euthanasia, navigating consultation requirements, and the roles of providers, patients, and their relatives (which are governed by all six sources of regulation).

Of note were two specific applications of the euthanasia law which were the subject of more detailed regulation than more general euthanasia practice in terms of the number and type of sources of regulation: advance directives (in terms of identifying and managing a patient’s advance request for euthanasia) and euthanasia requests based on mental illness (in terms of the duration of the euthanasia process in this case).

Finally, the results show that law governs all domains of euthanasia practice except one: support for health professionals who provide euthanasia care. Other sources of regulation govern this domain, guiding clinicians in providing ethical, professional, high-quality euthanasia care, and ensuring that providers are supported in providing euthanasia.

### Interpretation of Main Findings

The findings from this study demonstrate the complexity of the Act’s implementation into practice. The Act is very short and appears relatively straightforward by comparison with other assisted dying legislation internationally (Waller et al., 2023). Because of this, the law is silent on, or only partially addresses, many important parts of euthanasia practice, particularly clinical and practical considerations (Tack, 2009). Our findings suggest that this required regulatory guidance has been provided by other sources of regulation. Organisations have moved to fill these regulatory vacuums and address otherwise unregulated issues, by developing guidance to support the implementation of the Act into their own activities and operations (Tack, 2009; Van Wesemael, Cohen, Onwuteaka-Philipsen, Bilsen, Distelmans, et al., 2009).

As noted above, some domains of euthanasia practice, such as advance directives and euthanasia requests based on mental illness, are more ‘heavily’ regulated. By this we mean both that they are governed by a greater breadth of sources of regulation and by a larger number of regulatory instruments. Possibly, this reflects the role and limits of law in guiding behaviour. Regulatory scholarship supports the *utility* of law in establishing broad parameters of a system but recognises its *limitations* in providing sufficiently detailed and nuanced guidance for implementation into clinical practice (Scott, 2001; White et al., 2022). The limitations of the law are particularly apparent where complex clinical-ethical discretion or judgement must be exercised. Certainly, the literature supports the important role for non-legal regulation to guide decision-making when assessing patients with mental illness for euthanasia (Verhofstadt et al., 2019, 2020, 2021). International evidence also supports this interpretation of the role and limits of assisted dying laws; studies on Victorian (Australia) and Canadian assisted dying regulation show the important role played by non-legal sources such a policy in guiding practice (Close et al., 2021; Silvius et al., 2019) and by clinical education in translating the law into practice for clinicians (Brown et al., 2020; White et al., 2021).

This is not necessarily a criticism of the Belgian law. Indeed, the authority of the law is required to permit euthanasia to occur and draw essential boundaries for the legal framework, such as how euthanasia is defined and who is eligible for euthanasia. But different sources of regulation may be better suited for particular purposes, especially in relation to some clinical and practical areas of euthanasia practice (Black, 2002; White et al., 2022). In this way, non-legal sources of regulation might attempt to move the boundaries established by law. Sometimes, these attempts have their intended effect. For example, health care organisations and institutions move the boundaries of practice by tailoring the legal requirements to their local circumstances (Lemiengre et al., 2010). In other cases, these attempts do not have their intended effect, and the lack of clear legal direction on a particular issue seems to be a practical barrier to its occurrence. For example, the law itself is silent on PAS. While non-legal sources of regulation (the CFCEE and national medical association) endorse the permissibility of PAS (CFCEE, 2005; Nationale Raad Van De Orde Der Geneesheren, 2004) the permissibility of PAS remains unclear and there is an overall low rate of PAS in Belgium (CFCEE, 2022).

### Implications for Practice, Policy, and Research

Findings about aspects of euthanasia practice being subject to multiple sources of regulation point to potential risks of inconsistent care provision. Where regulation is duplicated or conflicting, this could produce differences in patients’ experiences and unfairness or discrimination in terms of equity of access. While there is some evidence of variation in euthanasia practice across Belgium (Cohen et al., 2012; Lemiengre et al., 2008; Verhofstadt et al., 2020), the extent to which regulation is responsible for this is not clear and further research is needed. It is also not clear how to balance consistency and equity for patients on the one hand, and the preferences and capabilities of providers on the other (Ciornei et al., 2022; Gastmans, Lemiengre, & de Casterlé, 2006).

In addition, providers may experience confusion or frustration where the same domains of euthanasia practice are governed by multiple sources of regulation (and this regulation is inconsistent or contains differing emphases). To illustrate, we identified that four sources of regulation (law, policy, professional standards, and advisory documents) govern the duration of the euthanasia process for patients whose request is based on mental illness, as ranging from one-month, to six months, or one year (Verhofstadt et al., 2019, 2020). It is incumbent on policymakers to be aware of the fragmented nature of this wider regulatory landscape, so that, where possible, their guidelines might align with others, and avoid conflicts (White et al., 2022). A harmonised approach to regulating euthanasia across all sources of regulation is likely to promote consistency of practice, but it is unclear which organisation would have responsibility for overseeing this.

Finally, this study has implications for how researchers might map, examine, and compare the domains of euthanasia practice governed in assisted dying regulatory frameworks internationally. We hope that other researchers might adopt and refine this framework with a view to developing a more systematic understanding of the sources of regulation operating within assisted dying frameworks, and the domains of practice that they govern. We also anticipate that this research may facilitate international comparative work.

### Strengths and Weaknesses

This study drew on existing scholarship to undertake the first mapping of the domains of euthanasia practice that are governed by regulation in Belgium, advancing a holistic understanding of euthanasia regulation. It also has broader significance as a novel framework for analysis internationally. While scoping reviews place parameters on the extent of searching and analysis, a rigorous methodological approach was applied in this study, and a large number of records were reviewed for inclusion.

A potential limitation of this study is that it only identified the domains of euthanasia practice that the literature explicitly described as being governed by regulation. As such, certain domains may have been missed if not described in the literature. However, this is unlikely, as given their breadth, the domains of euthanasia practice identified in this study are likely to be comprehensive.

## Conclusion

This scoping review analysed the literature on the sources of regulation operating in the Belgium euthanasia system, and mapped the domains of euthanasia they each govern. Six broad domains of euthanasia practice were identified, incorporating 17 sub-domains. Multiple sources of regulation provide guidance to euthanasia providers on many parts of euthanasia practice. Overlap between regulatory guidelines has implications for patients being able to access euthanasia equitably, and for the consistency and quality of patient euthanasia care and may also complicate decision-making for providers. Policymakers in Belgium and internationally should aim for an integrated approach to regulation which harmonises existing ‘fragmented’ regulatory guidance.

## Supplemental Material

Supplemental material - What Domains of Belgian Euthanasia Practice are Governed and by Which Sources of Regulation: A Scoping Review Supplemental material for What Domains of Belgian Euthanasia Practice are Governed and by Which Sources of Regulation: A Scoping Review by Madeleine Archer, Lindy Willmott, Kenneth Chambaere, Luc Deliens, and Ben P. White in Journal of Death and Dying.
