# Supplementary material for: What Domains of Belgian Euthanasia Practice are Governed and by Which Sources of Regulation: A Scoping Review
Source: Omega (Westport). 2023 Dec 14;92(4):2105–30. doi: 10.1177/00302228231221839 (PMC12891245; doi:10.1177/00302228231221839)
Supplement: Supplemental material - What Domains of Belgian Euthanasia Practice are Governed and by Which Sources of Regulation: A Scoping Review [file sj-pdf-1-ome-10.1177_00302228231221839.pdf]

Supplementary file 1

<u>Governed domains of euthanasia practice (scoping review) protocol</u>

Objectives of the study

- To generate themes which reflect the domains of euthanasia practice governed by sources of regulation operating in the Belgian euthanasia regulatory system
- To map which sources of regulation govern which domains of practice

Research question(s)

- What are the domains of euthanasia practice that the literature identifies as being governed by Belgian euthanasia regulation?
- Which sources of regulation (as identified in the mapping review) govern which domains of euthanasia practice?

Key concepts

- Regulation
  - ‘Regulation is the sustained and focused attempt to alter the behaviour of others according to defined standards or purposes with the intention of producing a broadly identifiable outcome or outcomes, which may involve mechanisms of standard-setting, information-gathering and behaviour-modification’ (Julia Black, ‘Critical Reflections on Regulation’ (2002) 27 Australian Journal of Legal Philosophy 1, 26).
  - This definition captures the multiplicity and plurality of regulatory sources including non-state actors (regulation is not confined to State actors)
  - Conveys intentionality in regulatory sources having to deliberately attempt to impact decision-making about AD
  - Regulation occurs through a variety of sources e.g. law, policy, ethics, training, professional standards, etc
  - Our conception of regulation includes understanding both how the practice is regulated on paper and how regulation works in practice (both are relevant to identify how people make decisions about euthanasia). How the system is implemented has regulatory implications (influences decision-making), as do the support systems that are provided.

- Source of regulation (definitions and decisions used to identify these in the mapping review)
    - As informed by the adopted definition of regulation.
    - In order to be a source of regulation the person/group/thing/instrument must intend, in a sustained and focused way, to influence the practice of euthanasia in Belgium.
    - Practically, because intention is subjective etc, if a person/group/thing/instrument says/does something to others about how euthanasia should operate = source of regulation.
    - Sources of regulation, following on from definition of 'regulation' can be both State or non-State based.
    - e.g. 'culture' does not intend, in a sustained and focused way, to influence decision-making about euthanasia.
    - Note: Blanket exclusion of non-Belgian regulation (e.g. European, international) to focus in on specific, operational regulation within Belgium (02/03/22).
    - Note: Exclusion of individual actors (includes individuals, advance directives), consistent with this paper's definition of regulation given 'sustained and focused' requirement (02/03/22) and focus on structures and systems rather than e.g. advocacy efforts (01/10/22).
- Source of regulation (as identified in the mapping review)
    - Law
    - Policy
    - Professional standards
    - Training programs
    - Advisory documents
    - System design
- Regulatory functions that sources of regulation have (as identified in the mapping review)
    - Prescribing conduct
    - Scaffolding to support practice
    - Monitoring the system
- Euthanasia

- Includes voluntary euthanasia and physician assisted suicide. As defined/legalised by the Belgian Act on Euthanasia (active ending of life at the explicit request of the patient)(excl. involuntary and non-voluntary forms).

Broad scoping review method

Guided by Arksey and O'Malley's five-stage scoping review methodological framework (Hilary Arksey and Lisa O'Malley, 'Scoping Studies: Towards a Methodological Framework' (2005) 8(1) International Journal of Social Research Methodology 19).

- Stage 1: identify the research question
- Stage 2: identify relevant studies
- Stage 3: selects the studies
- Stage 4: charts the data
- Stage 5: collates, summarises and reports the results

Review inclusion criteria

- Journal article in a peer reviewed journal (or book chapter (25/03/22)).
- Full text available/able to be obtained, (if no abstract, take inclusive approach and proceed to full-text review).
- 28 May 2002 (inclusive) to present (to align with the research question capturing *current* sources of regulation, 2002 was the year the law was passed, not looking at euthanasia prior to the law, only regulation since the law).
- In English (search expanded to Dutch and French search terms on 09/03/22).
- <u>Belgian</u> <u>regulation</u> of <u>euthanasia</u> is a substantive focus of the paper

Review search strategy

- Databases scoped: EBSCO host (searching Psych Info, Legal Source, Cinahl, Medline), Scopus, Pubmed.
    - Rationale for inclusion/exclusion of databases. Few relevant articles/already captured in other databases: Web of Science, Embase, Westlaw International, Ageline, Social Work Abstracts. Inability to make consistent search terms in Google Scholar and HeinOnline (vast majority of articles already captured in HeinOnline.

    - Each of the included databases included some unique articles not already captured. Cover law and health papers.
- Search strategy is to target 2 criteria and a number of variations thereupon: (1) euthanasia and (2) Belgium within the title or the abstract of the paper
    - Euthanasia: Euthanasia; Voluntary euthanasia; Assisted dying; Assisted suicide; Assisted death; Physician assisted suicide; Physician assisted death; Physician assisted dying; Mercy killing; Medical assistance in dying; Medical aid in dying
    - Belgium: Belg* (covers Belgian, Belgium); Flemish; Flanders; Walloon; Wallonia; Benelux
- Limiters/filters in databases
    - Type of document: journal
    - 2002-present
    - Peer reviewed (if the option presents)
    - Language – English, French, Dutch
- Notes:
    - Searching periods will be limited.
    - These methods will be developed over the course of the study.
- Supplement the review sample with reference list searching (from already included records)

Specific scoping review method

This part of the method commences once all databases have produced a list of papers containing the results of the search strategy. Zotero will be used to collate and remove duplicates.

- First: pilot this protocol with 20 abstracts
- Remove duplicates.
- <u>Title screening.</u> Inclusion criteria applied to title. Inclusive approach will be applied due to limited information contained in title. Remove clearly irrelevant papers e.g. relate to euthanasia of animals (N = X) AND papers which have slipped through but which do not meet inclusion criteria e.g. are in another language, not the journal format specified in the inclusion criteria, etc.

- Abstract screening. Inclusion criteria applied to abstract. This is to be a robust screening stage but still take an inclusive approach. In order to satisfy 'substantive discussion of regulatory source', abstract must demonstrate this (show that a regulatory source will be substantively discussed/analysed or the paper is about analysing a regulatory source (N = X). Not constrained in this process by where in the abstract the inclusion criteria are met i.e. whether it is the objective of a paper or in its findings. An inclusive approach will be applied in this phase.
- Two authors to co-assess 30 abstracts and then lead author to complete the sample.
- Moderation to be completed blind to each moderator's thoughts, and disagreements to be resolved by discussing and reaching a view.
- Full-text screening. Inclusion criteria applied to full-text paper. Where a full-text reviewed paper meets the inclusion criteria, its references will be scanned and any relevant papers it produces will be subject to the above processes and may be included (N = X).
- Two authors to moderate a sample of 10 records using the same moderation processes as above.
- Final sample included in the review (N = X)

Data extraction

Revised during screening and full-text review.

Lead author first to familiarise with all articles by reading in full (without any data extraction)

Data to extract (unless adapted):

- Paper title/reference
- Type of study/paper (e.g. empirical)
- Focus of article (descriptive, e.g. hospital policies on euthanasia in Flanders)
- Source(s) of regulation (e.g. Act on euthanasia, Flemish nursing home policies on euthanasia or reported on a more macro level)
- Who is responsible for/makes the source of regulation (e.g. Ministry of Justice, Caritas Catholica)

- What domains of practice are governed by each source of regulation (extracted verbatim or in truncated version).
- Notes/regulatory observations

Data analysis

- The coding frame, chart, or 'template' used to house the extracted data to facilitate data analysis.
- In this study, only the descriptive record data and data on the domains of practice governed by each source of regulation is being analysed.
- Thematic analysis of this data as per 'template analysis' a 'codebook' approach to thematic analysis.
- Descriptive themes reflect the domains of euthanasia practice governed by regulation in Belgium.
- Analysis of descriptive data to produce descriptive statistics for the records included in the review.

Results

- Descriptive statistics
- Presentation on themes which describe the governed domains of euthanasia practice.

Supplementary file 2

Complete method: data collection

Data collection

A systematic search was undertaken for book chapters and journal articles in peer reviewed journals in six interdisciplinary databases, which were Scopus, Pubmed, Cinahl, Medline, Legal Source, and PsychInfo. Note that Cinahl, Medline, Legal Source, and PsychInfo were searched using a central EBSCOhost search that included results from each of these four databases.

The search strategy was applied to all records' abstracts and titles. The search strategy contained one term pertaining to assisted dying and another to Belgium, with variations in terminology and language (See Table A).

Table A: English, French, and Dutch database search terms

Criterion	English	French	Dutch
Assisted dying	euthanasia or voluntary euthanasia or assisted dying or assisted suicide or assisted death or physician assisted death or physician assisted dying or mercy killing or medical assistance in dying or medical aid in dying	euthanasie or aide medicale a mourir or aide au suicide or aider a mourir	euthanasie
Belgium	belg* or Flemish or Flanders or Walloon or Wallonia or benelux	belg* or flamand or flandre or wallonie or benelux	belg* or vlaam or vlaanderen or waals or wallonie or benelux

For the full search strategies and further information relating to the searches see below in this document under 'search strategy information.' The reference lists and footnotes of included records were also searched for relevant records not already identified, which met formal inclusion criteria (discussed below) and which explicitly concerned assisted dying in Belgium. Records were managed in Zotero.

Records identified from the database searching and reference lists were subject to title, abstract and full-text screening for eligibility, whereby all records were assessed against the inclusion criteria (discussed below). The screening process was moderated by [first author] and [last author] and was undertaken blind. Records progressed to the final sample where they met substantive inclusion criteria: they considered regulation in a substantive way, and engaged with one or more specific forms of regulation operating in the Belgian AD legal framework. Records were also required to meet formal inclusion criteria: the record was a book chapter or journal article in a peer reviewed journal, the full text of the record was available, published between 28 May 2002 (inclusive, as the date that the *Act* was enacted) to 2022 (the date the relevant search was undertaken)(see below), and written in English, Dutch, or French. The searching and screening processes resulted in a review sample which was then subject to data extraction and analysis.

Search strategy information

Three separate searches in the databases were undertaken

(1) Initial database search to capture only English journal articles
(2) Expansion of initial search to capture Dutch and French journal articles
(3) Expansion of initial search to capture book chapters in English

The details for each search are provided below, including the date on which the relevant search was undertaken.

Initial Database Search

Search undertaken 11/02/22

Database	Search Strategy	Limiters
EBSCOhost (Legal Source, APAPsychInfo, Medline, Cinahl)	S1: AB (euthanasia OR "voluntary euthanasia" OR "assisted dying" OR "assisted suicide" OR "assisted death" OR "physician assisted suicide" OR "physician assisted death" OR "physician assisted dying" OR "mercy killing" OR "medical assistance in dying" OR "medical aid in dying") AND AB (belg* OR flemish OR flanders OR walloon OR wallonia OR benelux)<br><br>S2: TI (euthanasia OR "voluntary euthanasia" OR "assisted dying" OR "assisted suicide" OR "assisted death" OR "physician assisted suicide" OR "physician assisted death" OR "physician assisted dying" OR "mercy killing" OR "medical assistance in dying" OR "medical aid in dying") AND TI (belg* OR flemish OR flanders OR walloon OR wallonia OR benelux)<br><br>S1 OR S2	From 2002-2022<br><br>Peer reviewed<br><br>Academic journals<br><br>Dutch; Flemish; English; French
Scopus	(TITLE ((euthanasia OR "voluntary euthanasia" OR "assisted dying" OR "assisted suicide" OR "assisted death" OR "physician assisted suicide" OR "physician assisted death" OR "physician assisted dying" OR "mercy killing" OR "medical assistance in dying" OR "medical aid in dying") AND (belg* OR flemish OR flanders OR walloon OR wallonia OR benelux))) OR (ABS ((euthanasia OR "voluntary euthanasia" OR "assisted dying" OR "assisted suicide" OR "assisted death" OR "physician assisted suicide" OR "physician assisted death" OR "physician assisted dying" OR "mercy killing" OR "medical assistance in dying" OR "medical aid in dying") AND (belg* OR flemish OR flanders OR walloon OR wallonia OR benelux)))	2002-2022<br><br>Article<br><br>Source type: Journal English, French, Dutch (exclude Spanish)
Pubmed	(euthanasia[Title/Abstract] OR "voluntary euthanasia"[Title/Abstract] OR "assisted dying"[Title/Abstract] OR "assisted suicide"[Title/Abstract] OR "assisted death"[Title/Abstract] OR "physician assisted suicide"[Title/Abstract] OR "physician assisted death"[Title/Abstract] OR "physician assisted dying"[Title/Abstract] OR "mercy killing"[Title/Abstract] OR "medical assistance in dying"[Title/Abstract] OR "medical aid in dying"[Title/Abstract]) AND (belg*[Title/Abstract] OR flemish[Title/Abstract] OR flanders[Title/Abstract] OR walloon[Title/Abstract] OR wallonia[Title/Abstract] OR benelux[Title/Abstract])	2002-2022<br><br>Dutch, English, French

For example, the search strategy applied in Pubmed was:

(euthanasia[Title/Abstract] OR "voluntary euthanasia"[Title/Abstract] OR "assisted dying"[Title/Abstract] OR "assisted suicide"[Title/Abstract] OR "assisted

death"[Title/Abstract] OR "physician assisted suicide"[Title/Abstract] OR "physician assisted death"[Title/Abstract] OR "physician assisted dying"[Title/Abstract] OR "mercy killing"[Title/Abstract] OR "medical assistance in dying"[Title/Abstract] OR "medical aid in dying"[Title/Abstract]) AND (belg*[Title/Abstract] OR flemish[Title/Abstract] OR flanders[Title/Abstract] OR walloon[Title/Abstract] OR wallonia[Title/Abstract] OR benelux[Title/Abstract])

Limiters were applied to this search:

- Year of publication: 2002-2022
- Journal articles in English, Dutch, and French

<u>Second Database Search</u>

Extension: to specifically capture French and Dutch journal articles

Search undertaken 09/03/22

**Database**	**Search Strategy**	**Limiters**
EBSCOhost (Legal Source, APAPsychInfo, Medline, Cinahl)	French title search (and same for abstract): euthanasie OR “aide medicale a mourir” OR “aide au suicide” OR “aider a mourir” AND belg* OR flamand OR flandre OR wallonie OR Benelux<br><br>Dutch title search (and same for abstract): euthanasie AND belg* OR vlaam OR vlaanderen OR waals OR wallonie OR benelux	2002-2022, Academic journal, French<br><br>2002-2022, Academic journal, Dutch/Flemish
Scopus	French<br>(TITLE ((euthanasie OR "aide medicale a mourir" OR "aide au suicide" OR "aider a mourir") AND (belg* OR flamand OR flandre OR wallonie OR benelux))) OR (ABS ((euthanasie OR "aide medicale a mourir" OR "aide au suicide" OR "aider a mourir") AND (belg* OR flamand OR flandre OR wallonie OR benelux)))<br><br>Dutch<br>(TITLE ((euthanasie) AND (belg* OR vlaam OR vlaanderen OR waals OR wallonie OR benelux))) OR (ABS ((euthanasie) AND (belg AND *or AND vlaam OR vlaanderen OR waals OR wallonie OR benelux)))	2002-2022, Journal article, French<br><br>2002-2022, Dutch, Source type: journal
Pubmed	French<br>(euthanasie[Title/Abstract] OR "aide medicale a mourir"[Title/Abstract] OR "aide au	2002-2022, French

	suicide"[Title/Abstract] OR "aider a mourir"[Title/Abstract]) AND (belg*[Title/Abstract] OR flamand[Title/Abstract] OR flandre[Title/Abstract] OR wallonie[Title/Abstract] OR benelux[Title/Abstract])<br><br>Dutch<br>(euthanasie[Title/Abstract]) AND (belg*[Title/Abstract] OR vlaam[Title/Abstract] OR vlaanderen[Title/Abstract] OR waals[Title/Abstract] OR wallonie[Title/Abstract] OR benelux[Title/Abstract])	2002-2022, Dutch

Third database search

Extension: to capture book chapters in English

Search undertaken on 25/03/22

**Database**	**Search Strategy**	**Limiters**
EBSCOhost (Legal Source, APAPsychInfo, Medline, Cinahl)	S1 or S2<br><br>S1 = AB (euthanasia OR "voluntary euthanasia" OR "assisted dying" OR "assisted suicide" OR "assisted death" OR "physician assisted suicide" OR "physician assisted death" OR "physician assisted dying" OR "mercy killing" OR "medical assistance in dying" OR "medical aid in dying") AND AB (belg* OR flemish OR flanders OR walloon OR wallonia OR benelux)<br><br>S2 = TI (euthanasia OR "voluntary euthanasia" OR "assisted dying" OR "assisted suicide" OR "assisted death" OR "physician assisted suicide" OR "physician assisted death" OR "physician assisted dying" OR "mercy killing" OR "medical assistance in dying" OR "medical aid in dying") AND TI (belg* OR flemish OR flanders OR walloon OR wallonia OR benelux)	2002-2022<br><br>Book
Scopus	(TITLE ((euthanasia OR "voluntary euthanasia" OR "assisted dying" OR "assisted suicide" OR "assisted death" OR "physician assisted suicide" OR "physician assisted death" OR "physician assisted dying" OR "mercy killing" OR "medical assistance in dying" OR "medical aid in dying") AND (belg* OR flemish OR flanders OR walloon OR wallonia OR benelux))) OR (ABS ((euthanasia OR "voluntary euthanasia" OR "assisted dying" OR "assisted suicide" OR "assisted death" OR "physician assisted suicide" OR "physician assisted death" OR "physician assisted dying" OR "mercy killing" OR "medical assistance in dying" OR "medical aid in dying") AND (belg* OR flemish OR flanders OR walloon OR wallonia OR benelux)))	2002-2022<br><br>Source type: book<br><br>Book chapter
Pubmed	(euthanasia[Title/Abstract] OR "voluntary euthanasia"[Title/Abstract] OR "assisted dying"[Title/Abstract] OR "assisted suicide"[Title/Abstract] OR "assisted death"[Title/Abstract] OR "physician assisted suicide"[Title/Abstract] OR "physician assisted	2002-2022<br><br>Books and documents

	death"[Title/Abstract] OR "physician assisted dying"[Title/Abstract] OR "mercy killing"[Title/Abstract] OR "medical assistance in dying"[Title/Abstract] OR "medical aid in dying"[Title/Abstract]) AND (belg*[Title/Abstract] OR flemish[Title/Abstract] OR flanders[Title/Abstract] OR walloon[Title/Abstract] OR wallonia[Title/Abstract] OR benelux[Title/Abstract])	

Supplementary file 3
Descriptive sample information

	Record citation	Record type	Language	Study type	Focus of record	Source(s) of regulation discussed
1	Nys, Herman, ‘A Discussion of the Legal Rules on Euthanasia in Belgium Briefly Compared with the Rules in Luxembourg and the Netherlands’ in David Albert Jones, Chris Gastmans and C MacKellar (eds), Euthanasia and Assisted Suicide: Lessons from Belgium (Cambridge University Press, 2018) 7	Book chapter	English	Non-empirical	Comparison of the Belgian, Dutch, and Luxembourg legal rules on euthanasia	Law, system design
2	Bosshard, G et al, ‘A Role for Doctors in Assisted Dying? An Analysis of Legal Regulations and Medical Professional Positions in Six European Countries’ (2008) 34(1) *Journal of Medical Ethics* 28	Article	English	Non-empirical	Examines the physician’s role in assisted dying in Western Europe by analysing legislation and medical professional positions	Law, professional standards
3	Murdoch, Joanna, ‘A Step Too Far or a Step in the Wrong Direction? A Critique of the 2014 Amendment to the Belgian Euthanasia Act’ (2021) 39(Suppl 1) *Monash Bioethics Review* 103	Article	English	Non-empirical	Explores the implications of the 2014 amendment to the Belgian Act	Law
4	Lewis, Penney and Isra Black, ‘Adherence to the Request Criterion in Jurisdictions Where Assisted Dying Is Lawful? A Review of the Criteria and Evidence in the Netherlands, Belgium, Oregon, and Switzerland’ (2013) 41(4) *Journal of Law, Medicine & Ethics* 885	Article	English	Non-empirical	Examines adherence to the legal criteria on requests for euthanasia in the Netherlands, Belgium, Oregon, and Switzerland	Law, system design
5	Herremans, Jacqueline, ‘[Advance directives: the legal situation in Belgium]’ [2008] (3) *Bulletin de la Societe des Sciences Medicales du Grand-Duche de Luxembourg* 305	Article	French	Non-empirical	Investigates the legal status of advance declarations (for euthanasia and other procedures) in Belgium	Law, professional standards, system design
6	Nys, Herman, ‘An Amendment of the Belgian Act on Euthanasia with Unintended and Undesirable Consequences’ (2021) 28(3) *European Journal of Health Law* 281	Article	English	Non-empirical	Discusses implications of the 2020 amendment to the Belgian Act	Law
7	Sercu, M et al, ‘Are General Practitioners Prepared to End Life on Request in a Country Where Euthanasia Is Legalised?’ (2012) 38(5) *Journal of Medical Ethics* 274	Article	English	Empirical	Qualitatively investigates how Flemish general practitioners deal with euthanasia	Law, professional standards, training, system design
8	Schweitser et al, 'Assessment of patient decision-making capacity in the context of voluntary euthanasia for psychic suffering caused by psychiatric disorders: a qualitative study of approaches among Belgian physicians' (2021) 47 *Journal of Medical Ethics* e38	Article	English	Empirical	Applied to the context of patients with psychiatric disorders, investigates how doctors assess decision-making capacity	Law, professional standards, system design
9	Paul Vanden Berghe et al, 'Assisted dying - The current situation in Flanders: Euthanasia embedded in palliative care' (2013) 20(6) *European Journal of Palliative Care* 266	Article	English	Non-empirical	Members of the Federation of Palliative Care Flanders describe the relationship between assisted dying and palliative care over the last 10 years	Law, policy, training, system design
10	Rory Watson, 'Assisted dying: Belgian doctors are acquitted of unlawful poisoning' (2020) 368 *BMJ Clinical Research Ed* m425	Article	English	Non-empirical	News report on a criminal case in Belgium regarding euthanasia (January 2020 in Ghent)	Law

11	Rory Watson, 'Belgian Doctors stand trial in landmark case' (2020) 368 *BMJ clinical research ed* m259	Article	English	Non-empirical	News report on a criminal case in Belgium regarding euthanasia (January 2020 in Ghent)	Law
12	Berghe, PV et al, ‘Assisted Dying: The Current Situation in Flanders: Euthanasia Embedded in Palliative Care’ in David Albert Jones, Chris Gastmans and Calum MacKellar (eds), *Euthanasia and Assisted Suicide: Lessons from Belgium* (Cambridge University Press, 2017) 67	Book chapter	English	Non-empirical	Federation of Palliative Care Flanders members describe the relationship between assisted dying and palliative care over the last 10 years, incorporating some recent updates	Law, system design
13	Smets, Tinne et al, ‘Attitudes and Experiences of Belgian Physicians Regarding Euthanasia Practice and the Euthanasia Law’ (2011) 41(3) *Journal of Pain and Symptom Management* 580	Article	English	Empirical	Examines of the attitudes of Belgian doctors towards the Belgian euthanasia legislation and the use of life-ending medications	Law, training, system design
14	Van Overstraeten, Marc, ‘Belgian Act on Euthanasia: Alterations to Be Expected?’ (2009) 28(4) *Medicine and Law* 755	Article		Non-empirical	Examines the impact of two constitutional court decisions and various proposed amendments to the 2002 Act on the Act and the legalised practice	Law, system design
15	Cohen-Almagor, R, ‘Belgian Euthanasia Law: A Critical Analysis’ (2009) 35(7) *Journal of Medical Ethics* 436	Article	English	Non-empirical	Critically analyses the Belgian law on euthanasia	Law, policy, training
16	Van Assche, Kristof et al, ‘“Capacity for Discernment” and Euthanasia on Minors in Belgium’ (2019) 27(2) *Medical Law Review* 242	Article	English	Non-empirical	Applies a critical analytical legal lens to the concept of 'capacity for discernment' and its practical and legal implications	Law, professional standards, system design
17	Lemmens, Trudo, ‘Charter Scrutiny of Canada’s Medical Assistance in Dying Law and the Shifting Landscape of Belgian and Dutch Euthanasia Practice’ (2018) 85 *Supreme Court Law Review* 459	Article	English	Non-empirical	Discusses the evidence that has emerged from Belgium and the Netherlands arguing that medical assistance in dying should be restricted to end-of-life situations	Law, policy, professional standards, training, advisory documents, system design
18	Adams, Maurice and Herman Nys, ‘Comparative Reflections on the Belgian Euthanasia Act 2002’ (2003) 11(3) *Medical Law Review* 353	Article	English	Non-empirical	Compares the Belgian and Dutch pieces of legislation and differentiates them in terms of their broader (mainly political) context	Law, professional standards, system design
19	Van Wesemael, Yanna et al, ‘Consulting a Trained Physician When Considering a Request for Euthanasia: An Evaluation of the Process in Flanders and the Netherlands’ (2010) 33(4) *Evaluation & The Health Professions* 497	Article	English	Empirical	Compares the characteristics of euthanasia consultations undertaken by SCEN in the Netherlands and LEIF in Belgium and evaluates consultation quality in each jurisdiction	Law, training
20	Lemiengre, Joke et al, ‘Content Analysis of Euthanasia Policies of Nursing Homes in Flanders (Belgium)’ (2009) 12(3) *Medicine, Health Care, and Philosophy* 313	Article	English	Empirical	Analyses the content of ethics policies on euthanasia in the Flemish nursing home setting, specifically considering how the religious affiliation of the institution impacts policy content	Law, policy, system design
21	Englert, M, ‘[Control of the Legal Practice of Euthanasia in Belgium]’ (2015) 36(1) *Revue Medicale de Bruxelles* 45	Article	French	Non-empirical	Analyses the workings of the Federal Control and Evaluation Commission and raises criticisms with respect to the committee’s quality and efficacy	Law, system design
22	Cohen, Joachim et al, ‘Cultural Differences Affecting Euthanasia Practice in Belgium: One Law but Different	Article	English	Empirical	Investigates cultural differences between euthanasia practice in different regions of Belgium	Law, training, system design

	Attitudes and Practices in Flanders and Wallonia' (2012) 75(5) *Social Science & Medicine (1982)* 845					
23	Englert, M, '[Depenalized Practice of Euthanasia in Belgium: Evolution from 2002 to 2005 and Interpretation of the Differences between the North and the South of the Country]' (2007) 28(5) *Revue Medicale de Bruxelles* 423	Article	French	Non-empirical	Examines Federal Control and Evaluation Commission biannual reports and examines differences between Flanders and Wallonia with respect to reported cases	Law, professional standards, training, system design
24	Englert, M, '[Depenalized Practice of Euthanasia in Belgium]' (2005) 60(4) R*evue Medicale de Liege* 227	Article	French	Non-empirical	Analyses and critiques the Federal Control and Evaluation Commission's first report	Law, professional standards, system design
25	Gastmans, Chris, Joke Lemiengre and Bernadette Dierckx de Casterlé, 'Development and Communication of Written Ethics Policies on Euthanasia in Catholic Hospitals and Nursing Homes in Belgium (Flanders)' (2006) 63(1–2) *Patient Education and Counseling* 188	Article	English	Empirical	Examines ethics policies on euthanasia in Catholic nursing homes in Flanders and the extent to which these policies have been communicated	Law, policy, professional standards
26	Bernheim, Jan L et al, 'Development of Palliative Care and Legalisation of Euthanasia: Antagonism or Synergy?' (2008) 336(7649) *BMJ (Clinical research ed.)* 864	Article	English	Non-empirical	Discusses the development of, and relationship between palliative care and euthanasia in Belgium	Law, policy, professional standards, training, system design
27	Friedel, Marie, 'Does the Belgian Law Legalising Euthanasia for Minors Really Address the Needs of Life-Limited Children?' (2014) 20(6) *International Journal of Palliative Nursing* 265	Article	English	Non-empirical	Analyses key aspects of the 2014 amendment to the Act in order to identify whether the new law really addresses the needs of life-limited children	Law, professional standards
28	Dierickx, Sigrid et al, 'Drugs Used for Euthanasia: A Repeated Population-Based Mortality Follow-Back Study in Flanders, Belgium, 1998-2013' (2018) 56(4) *Journal of Pain and Symptom Management* 551	Article	English	Empirical	Describes the medications used to perform euthanasia in Flanders and how these medications have changed over time	Law, professional standards, training, system design
29	Van Wesemael, Yanna et al, 'Establishing Specialized Health Services for Professional Consultation in Euthanasia: Experiences in the Netherlands and Belgium' (2009) 9 *BMC Health Services Research* 220	Article	English	Empirical	Describes and compares specialised services providing consultants as mandated by the respective Belgian and Dutch laws: SCEN and LEIF	Law, training
30	Lemiengre, Joke et al, 'Ethics Policies on Euthanasia in Hospitals--A Survey in Flanders (Belgium)' (2007) 84(2–3) *Health Policy* 170	Article	English	Empirical	Investigates institutional ethics policies on euthanasia in hospitals in Flanders	Law, policy, professional standards
31	Lemiengre, Joke et al, 'Ethics Policies on Euthanasia in Nursing Homes: A Survey in Flanders, Belgium' (2008) 66(2) *Social Science & Medicine* (1982) 376	Article	English	Empirical	Investigates ethics policies on euthanasia in Catholic nursing homes in Flanders	Law, policy, professional standards
32	Lossignol, D, '[Euthanasia 2002-2014: The Situation in Belgium]' (2016) 33(8) *Revue des Maladies Respiratoires* 692	Article	French	Non-empirical	Examines and evaluates the legal framework for assisted dying in Belgium to examine the legal situation, data, the federal control and evaluation commission, the legal conditions and concerns about practice	Law, professional standards, training, advisory documents, system design
33	Dom, Geert et al, 'Euthanasia and Assisted Suicide in the Context of Psychiatric Disorders: Sharing Experiences from the Low Countries' (2020) 54(4) *Psychiatria Polska* 661	Article	English	Non-empirical	Presents an overview and identifies challenges associated with the Belgian and Dutch experiences with euthanasia for people with psychiatric disorders	Law, professional standards, system design
34	Liégeois, A, 'Euthanasia and Mental Suffering: An Ethical Advice for Catholic Mental Health Services' (2013) 19(1) *Christian Bioethics* 72	Article	English	Non-empirical*	Provides an ethical advice on how Catholic mental health services should engage with euthanasia	Law, policy

35	Lossignol, D, '[Euthanasia and the Abuse of Conscience Clause Concept]' (2016) 37(4) *Revue Medicale de Bruxelles* 384	Article	French	Non-empirical	Presents an analysis of the Belgian Act with respect to conscientious objection	Law, professional standards, advisory documents, system design
36	Verhofstadt, Monica et al, 'Euthanasia in Adults with Psychiatric Conditions: A Descriptive Study of the Experiences of Belgian Psychiatrists' (2021) 104(3) *Science Progress* 368504211029775	Article	English	Empirical	Investigates the experience of psychiatrists who assess adults with psychiatric conditions for euthanasia	Law, professional standards, training, system design
37	Lewis, Penney, 'Euthanasia in Belgium Five Years after Legalisation' (2009) 16(2) *European Journal of Health Law* 125	Article	English	Non-empirical	Compares the Belgian, Dutch, and Luxembourg legal rules on euthanasia	Law, policy, professional standards, system design
38	Saad, Toni C, 'Euthanasia in Belgium: Legal, Historical and Political Review' (2017) 32(2) *Issues in Law & Medicine* 183	Article	English	Non-empirical	Considers Belgian euthanasia practice and policy before legalisation, the evolution of the Belgian Act, criticisms of the legislation, the influence of politics, and amendments to the Belgian Act	Law, system design
39	Raus, Kasper, Bert Vanderhaegen and Sigrid Sterckx, 'Euthanasia in Belgium: Shortcomings of the Law and Its Application and of the Monitoring of Practice' (2021) 46(1) *Journal of Medicine and Philosophy* 80	Article	English	Non-empirical	Presents a legal analysis of the shortcomings of the Act and the widening in practice of several legal requirements in the Act	Law, professional standards, system design
40	Smets, Tinne et al, 'Euthanasia in Patients Dying at Home in Belgium: Interview Study on Adherence to Legal Safeguards' (2010) 60(573) *British Journal of General Practice e163*	Article	English	Empirical	Explores the extent to which GPs adhere to the legal requirements for euthanasia in the context of patients at home, and seeks to identify some reasons for non-adherence	Law, professional standards, training, system design
41	Behaegel, J, S Vercoutere and D Matthys, 'Euthanasia in Psychiatric Patients' (2015) 71(17) *Tijdschrift voor Geneeskunde* 1086	Article	Dutch	Non-empirical	Applies the legal criteria of the Belgian Act to the context of patients with psychiatric disorders	Law, system design
42	Cleemput, Jasper and Birgitte Schoenmakers, 'Euthanasia in the Case of Dementia: A Survey among Flemish GPs' [2019] *BJGP open*	Article	English	Empirical	Investigates Flemish GPs' attitudes and understanding towards euthanasia for patients with dementia	Law
43	Nys, Herman, 'A Presentation of the Belgian Act on Euthanasia against the Background of Dutch Euthanasia Law' (2003) 10(3) *European Journal of Health Law* 239	Article	English	Non-empirical	Compares the Belgian and Dutch euthanasia laws	Law, professional standards, system design
44	Cohen-Almagor, Raphael, 'Euthanasia Policy and Practice in Belgium: Critical Observations and Suggestions for Improvement' (2009) 24(3) *Issues in Law & Medicine* 187	Article	English	Non-empirical	Identifies concerns with the Belgian Act, its interpretations and implementation, and developments since the law became operational	Law, professional standards, training
45	Nau, Jean-Yves, '[Euthanasia: A Few Belgian Points]' (2013) 9(396) *Revue Medicale Suisse* 1606	Article	French	Non-empirical	Examines the Federal Control and Evaluation Commission's response to a recent opinion of the French National Ethics Committee on the Belgian euthanasia law	Law, system design
46	Lossignol, D, '[Euthanasia: Status Report in 2014]' (2014) 35(4) *Revue Medicale de Bruxelles* 379	Article	French	Non-empirical	Gives a status report and evaluation of Belgian euthanasia practice in 2014	Law, professional standards, training, advisory documents, system design

47	Gastmans, C, F Van Neste and P Schotsmans, 'Facing Requests for Euthanasia: A Clinical Practice Guideline' (2004) 30(2) *Journal of Medical Ethics* 212	Article	English	Non-empirical*	Investigates how to translate legal rules on euthanasia into patient care	Law, policy, system design
48	Cohen-Almagor, Raphael, 'First Do No Harm: Pressing Concerns Regarding Euthanasia in Belgium' (2013) 36(5–6) *International Journal of Law and Psychiatry* 515	Article	English	Non-empirical	Documents concerns about the law	Law, training, system design
49	Van Humbeeck, Liesbeth et al, 'Flemish Healthcare Providers' Attitude towards Tiredness of Life and Euthanasia: A Survey Study' (2022) 26(1) *Aging & Mental Health* 205	Article	English	Empirical	Explores the legal understanding that doctors and nurses have about euthanasia in the context of older persons who are tired of life	Law, system design
50	Burette, Philippe et al, '[Four Years of Application of the Law That Decriminalizes Euthanasia in Belgium]' (2008) 37(9) *Presse Medicale* 1281	Article	French	Non-empirical	Analyses the content of the first two Federal Control and Evaluation Commission reports and interprets aspects of interest to the physician	Law, professional standards, training, system design
51	Verhofstadt, M et al, 'Ghent University Hospital's Protocol Regarding the Procedure Concerning Euthanasia and Psychological Suffering' (2019) 20(1) *BMC Medical Ethics* 59	Article	English	Non-empirical	Explains and examines Ghent University Hospital's policy on handling requests for euthanasia from patients who are external to the hospital and experiencing psychological suffering	Law
52	Lemiengre, Joke et al, 'How Do Hospitals Deal with Euthanasia Requests in Flanders (Belgium)? A Content Analysis of Policy Documents' (2008) 71(2) *Patient Education and Counseling* 293	Article	English	Empirical	Describes the nature and content of ethics policies on euthanasia in hospitals in Flanders and the impact of religious affiliation on policy content	Law
53	Van Wesemael, Yanna et al, 'Implementation of a Service for Physicians' Consultation and Information in Euthanasia Requests in Belgium' (2012) 104(3) *Health Policy* 272	Article	English	Empirical	Studies the implementation of LEIF	Law
54	Lemiengre, Joke et al, 'Institutional Ethics Policies on Medical End-of-Life Decisions: A Literature Review' (2007) 83(2–3) *Health Policy* 131	Article	English	Empirical	Examines the prevalence, content, communication, and implementation of ethics policies within institutions on medical end of life decisions	Law
55	Zeebroeck, Shanthi Van, 'Kill First, Ask Questions Later: The Rule of Law and the Belgian Euthanasia Act of 2002' (2018) 39(3) *Statute Law Review* 244	Article	English	Non-empirical	Critiques the Act and with reference to notions of the Rule of Law and legal certainty	Law
56	Veny, Ludo M, 'Law and Ethics. The Belgian Law on Euthanasia and Minors ... A Bridge Too Far for The Current Decade?' [2015] (1) *Fiat Iustitia* 197	Article	English	Non-empirical	Analyses the Belgian Act and the 2014 amendment to minors with the capacity for discernment	Law
57	Englert, M, '[Management of Requests for Euthanasia. Discussion of Cases]' (2008) 29(4) *Revue Medicale de Bruxelles* 429	Article	French	Non-empirical	Reviews the steps that a physician must follow in response to a patient's request for euthanasia. Discusses the interpretations of the legal conditions from reports of ten cases of assisted dying	Law, training, system design
58	Herremans, J and G Genicot, 'Minors in the Belgian Law on Euthanasia: A Welcomed Extension of Patient's Autonomy' (2016) 2016(3) *BioLaw Journal* 9	Article	English	Non-empirical	Argues that the 2014 extension of the Belgian Act is a positive extension of patients' autonomy	Law, system design
59	Cohen, Joachim et al, 'Nationwide Survey to Evaluate the Decision-Making Process in Euthanasia Requests in Belgium: Do Specifically Trained 2nd Physicians Improve Quality of Consultation?' (2014) 14 *BMC Health Services Research* 307	Article	English	Empirical	Assesses and compares the quality of euthanasia consultations in Flanders and Brussels (LEIF and non-LEIF physicians)	Law, training, system design

60	Bilsen, J et al, ‘Nurses’ Involvement in Physician-Assisted Dying under the Euthanasia Law in Belgium’ (2014) 51(12) *International Journal of Nursing Studies* 1696	Article	English	Empirical	Examines nurses' role and involvement in euthanasia in Belgium	Law, system design
61	Nys, Herman, ‘Physician Assisted Suicide in Belgian Law’ (2005) 12(1) *European Journal of Health Law* 39	Article	English	Non-empirical	Examines the legal status of assisted suicide in the Belgian euthanasia regime	Law
62	D’Haene, Ina et al, ‘Policies to Improve End-of-Life Decisions in Flemish Hospitals: Communication, Training of Health Care Providers and Use of Quality Assessments’ (2009) 8 *BMC Palliative Care* 20	Article	English	Empirical	Investigates the prevalence and nature of end-of-life policy implementation activities in acute hospitals in Flanders	Law, policy
63	Gastmans, Chris et al, ‘Prevalence and Content of Written Ethics Policies on Euthanasia in Catholic Healthcare Institutions in Belgium (Flanders)’ (2006) 76(2) *Health Policy* 169	Article	English	Empirical	Investigates ethics policies for competent terminally ill, incompetent terminally ill, and non-terminally ill patients in Catholic healthcare institutions in Belgium	Law, policy, professional standards, advisory documents, system design
64	Lemmens, W, ‘Psychiatric Patients and the Culture of Euthanasia in Belgium’ in David Albert Jones, Chris Gastmans and Calum MacKellar (eds), Euthanasia and Assisted Suicide: Lessons from Belgium (Cambridge University Press, 2017) 258	Book chapter	English	Non-empirical	Makes critical observations on Belgian euthanasia practice	Law
65	Verhofstadt, Monica et al, ‘Psychiatric Patients Requesting Euthanasia: Guidelines for Sound Clinical and Ethical Decision Making’ (2019) 64 *International Journal of Law and Psychiatry* 150	Article	English	Empirical	Studies several guidelines to identify proposed measures in them to operationalise the Belgian Act’s legal criteria, to identify suggestions for additional safeguards going beyond the legal criteria and to identify remaining fields of tension	Law, policy, professional standards, training, advisory documents, system design
66	Ducatelle, C et al, ‘Psychiatry and Euthanasia in Belgium, within the International Context’ (2005) 61(2) *Tijdschrift voor Geneeskunde* 83	Article	Dutch	Non-empirical	Provides a picture of the current relationship between psychiatry and euthanasia in Belgium, to compare it with the international situation and to highlight the main points of discussion in the literature on the subject	Law, policy, training
67	Bernheim, Jan et al, ‘Questions and Answers on the Belgian Model of Integral End-of-Life Care: Experiment? Prototype?’ (2014) 11(4) *Journal of Bioethical Inquiry* 507	Article	English	Non-empirical	Argues for a complementary and synergistic relationship between euthanasia and palliative care in Belgium	Law, policy, training
68	Nys, Herman, ‘Recent Developments in Health Law in Belgium’ (2006) 13(2) *European Journal of Health Law* 95	Article	English	Non-empirical	Describes recent developments in health law in Belgium, including those on euthanasia	Law
69	Lewis, Penney and Isra Black, ‘Reporting and Scrutiny of Reported Cases in Four Jurisdictions Where Assisted Dying Is Lawful: A Review of the Evidence in the Netherlands, Belgium, Oregon and Switzerland’ (2013) 13(4) *Medical Law International* 221	Article	English	Non-empirical	Examines assisted dying reporting and scrutiny mechanisms and legislation in the Netherlands, Belgium, Oregon, and Switzerland	Law, professional standards, system design
70	Smets, Tinne et al, ‘Reporting of Euthanasia in Medical Practice in Flanders, Belgium: Cross Sectional Analysis of Reported and Unreported Cases’ (2010) 341 *BMJ (Clinical research ed.)* c5174	Article	English	Empirical	Estimates the reporting rates of euthanasia to the Federal Commission and undertakes a comparison of reported and unreported cases	Law, training, system design
71	Nys, H, ‘Rights and Duties of Physicians under the Belgian Law on Euthanasia’ (2004) 60(3) *Tijdschrift voor Geneeskunde* 182	Article	Dutch	Non-empirical	Presents a legal analysis of the Act on euthanasia from the perspective of the physician confronted with a euthanasia	Law, system design

					request, and presents a manual for how to handle requests legally in a careful manner	
72	Van Wesemael, Yanna et al, ‘Role and Involvement of Life End Information Forum Physicians in Euthanasia and Other End-of-Life Care Decisions in Flanders, Belgium’ (2009) 44(6) *Health Services Research* 2180	Article	English	Empirical	Describes the role and involvement of LEIF doctors in end-of-life care decisions and euthanasia in Flanders	Law, training
73	Gastmans, Chris, Joke Lemiengre and Bernadette Dierckx de Casterlé, ‘Role of Nurses in Institutional Ethics Policies on Euthanasia’ (2006) 54(1) *Journal of Advanced Nursing* 53	Article	English	Empirical	Investigates ethics policies on euthanasia in Flemish Catholic healthcare institutions and identifies nurses’ roles within these policies	Law, policy, professional standards
74	Cohen-Almagor, Raphael, ‘Should the Euthanasia Act in Belgium Include Minors?’ (2018) 61(2) *Perspectives in Biology and Medicine* 230	Article	English	Non-empirical	Critically analyses the 2014 amendment to the Belgian Act regarding the extension to minors with capacity for discernment	Law, system design
75	Deliens, L and G van der Wal, ‘[Similarities and Differences between the Euthanasia Laws in Belgium and the Netherlands]’ (2003) 147(4) *Nederlands Tijdschrift voor Geneeskunde* 169	Article	Dutch	Non-empirical	Examines, compares, and contrasts assisted dying laws in Belgium and the Netherlands	Law, training, system design
76	Adams, Maurice and Heleen Weyers, ‘Supervision and Control in Euthanasia Law: Going Dutch?’ (2012) 23(2) *King’s Law Journal* 121	Article	English	Non-empirical	Examines how the assisted dying supervision and control systems in Belgium and the Netherlands are organised	Law, training, system design
77	Demedts, Dennis et al, ‘The Attitudes, Role & Knowledge of Mental Health Nurses towards Euthanasia Because of Unbearable Mental Suffering in Belgium: A Pilot Study’ (2018) 25(7) *Journal of Psychiatric and Mental Health Nursing* 400	Article	English	Empirical	Investigates the attitudes, roles, and knowledge of mental health nurses regarding euthanasia for patients with unbearable psychological suffering	Law, policy, professional standards, training
78	Montero, E, ‘The Belgian Experience of Euthanasia since Its Legal Implementation in 2002’ in David Albert Jones and Calum MacKellar (eds), Euthanasia and Assisted Suicide: Lessons from Belgium (Cambridge University Press, 2017) 26	Book chapter	English	Non-empirical	Investigates the legal status of euthanasia since 2002 and explores whether the strict legal criteria in the law have been rigorously respected, such that this practice can be considered ‘under control’	Law, policy, professional standards, training, system design
79	Verhofstadt, Monica et al, ‘The Engagement of Psychiatrists in the Assessment of Euthanasia Requests from Psychiatric Patients in Belgium: A Survey Study’ (2020) 20(1) *BMC Psychiatry* 400	Article	English	Empirical	Investigates why and how psychiatrists become engaged in assessing patients with psychiatric conditions who request euthanasia	Law, policy, professional standards, advisory documents, system design
80	Deliens, L, JL Bernheim and G Van Der Wal, ‘The Euthanasia Laws in Belgium and the Netherlands: A Comparison’ (2003) 58(7–8) *Revue Medicale de Liege 485*	Article	English	Non-empirical	Compares and contrasts the assisted dying legislation in Belgium and the Netherlands	Law, professional standards, system design, training
81	Raus, Kasper, ‘The Extension of Belgium’s Euthanasia Law to Include Competent Minors’ (2016) 13(2) *Journal of Bioethical Inquiry* 305	Article	English	Non-empirical	Describes how the 2014 amendment to the law came to be passed and identifies and critiques arguments for and against the law	Law, system design
82	Vanderhaegen, B, ‘The Inclined Plane Is a Fact: Evaluation of a Decade Practicing Euthanasia in Belgium’ (2014) 24(4) *Ethische Perspectieven* 307	Article	Dutch	Non-empirical	Reflects on the law so far and indicates emerging trends	Law, policy, training, system design
83	Smets, Tinne et al, ‘The Labelling and Reporting of Euthanasia by Belgian Physicians: A Study of Hypothetical Cases’ (2012) 22(1) *European Journal of Public Health* 19	Article	English	Empirical	Examines how doctors understand and label end-of-life decisions in Belgium and their attitudes towards this labelling	Law, training, system design

84	Smets, Tinne et al, 'The Medical Practice of Euthanasia in Belgium and The Netherlands: Legal Notification, Control and Evaluation Procedures' (2009) 90(2–3) *Health Policy* 181	Article	English	Non-empirical	Describes and compares current mechanisms for euthanasia oversight in Belgium and the Netherlands	Law, training, system design
85	Gamester N and Van den Eynden B, 'The Relationship between Palliative Care and Legalized Euthanasia in Belgium' (2009) 12(7) *Journal of Palliative Medicine* 589	Article	English	Non-empirical	Criticises the view that euthanasia and palliative care are integrated and synergistic	Law, training
86	Inghelbrecht, Els et al, 'The Role of Nurses in Physician-Assisted Deaths in Belgium' (2010) 182(9) *CMAJ* 905	Article	English	Empirical	Examines nurses' involvement in decision-making about euthanasia and the technical performance of euthanasia	Law, system design
87	Broeckaert, Bert, 'Treatment Decisions in Advanced Disease: A Conceptual Framework.' (2009) 15(1) *Indian Journal of Palliative Care* 30	Article	English	Non-empirical*	Categorises and distinguishes a number of treatment options that can be available in late stages of advanced disease	Law, policy
88	Englert, M, B Hanson and D Lossignol, '[Two Years of Legal Practice of Euthanasia in Belgium: Comparison with the Netherlands. First Evaluation in a Palliative Care Unit]' (2005) 26(3) *Revue Medicale de Bruxelles* 145	Article	French	Non-empirical	Comments on the data arising from the Federal Control and Evaluation Commission's first report and compares it with the data in the Netherlands. Illustrates the first two years of the law's application by examining the experience of a supportive and palliative care unit	Law, professional standards, system design
89	Lemiengre, Joke et al, 'Written Institutional Ethics Policies on Euthanasia: An Empirical-Based Organizational-Ethical Framework' (2014) 17(2) *Medicine, Health Care, and Philosophy* 215	Article	English	Empirical	Investigates healthcare organisations' implementation of euthanasia through written ethics policies	Law, policy
90	Dewallens, F and T Vansweevelt, 'Hoofdstuk II. Euthanasie' in F Dewallens and T Vansweevelt (eds), Handboek gezondheidsrecht Volume II (Intersentia, 2014) 1319	Book chapter	Dutch	Non-empirical	Provides a detailed overview of the assisted dying legal framework in Belgium	Law, professional standards, system design
91	Balthazar, Tom, 'Rapportering en control na euthanasie' in M Cosyns et al (eds), De euthanasiewet doorgelicht (Kluwer, 2003) 151	Article	Dutch	Non-empirical	Describes the nature of the control system of assisted dying in Belgium, and compares it with the Dutch system	Law, professional standards, system design
92	Broekaert, B and R Janssens, 'Palliative Care and Euthanasia: Belgian and Dutch Perspectives' in P Schotsmans and T Meulengergs (eds), Euthanasia and Palliative Care in the Low Countries (Peeters, 2005) 35	Book chapter	English	Non-empirical	Describes the role and involvement of palliative care organisations in euthanasia law reform in the Netherlands and Belgium	Law, policy, professional standards
93	Claes, Stephan et al, 'Euthanasia for Psychiatric Patients: Ethical and Legal Concerns about the Belgian Practice' [2015] (November) *BMJ Open* 4	Article	English	Non-empirical	Comments on another paper in BMJ open by Thienpont and colleagues, and argues that this study denies several important and unresolved issues in this field	Law, system design
94	D'Haene, Ina and Ina Pasman, 'Stand ban zaken intellingsbeleif' in Palliatieve zorg en euthanasie in Belgie. Evaluatie van de praktijk en de wetten (ASP, 2011) 268	Book chapter	Dutch	Empirical	Discusses the presence, content, and publication of the policy on euthanasia in various types of institutions for intramural healthcare in Flanders	Law, policy, system design
95	De Bondt, W, 'De eerste Evaluatie van de Toepassing van de Euthanasiewet: Capita selecta en Kanttekeningen' (2005-2006) 3 *Rechtskundig Weekblad* 81	Article	Dutch	Non-empirical	Clarifies and annotates the data in the Federal Control and Evaluation Commission's first report	Law, training, system design
96	Delbeke, E, 'Euthanasie bij minderjarigen' ['Euthanasia on Minors'] (2015) 3 *Tijdschrift voor Gezondheidsrecht* 163	Article	Dutch	Non-empirical	Critically examines the stricter approach to access to assisted dying taken in the 2014 amendment of the law to minors	Law, professional standards, system design

97	Delbeke, Evelien, 'Wettelijk Kader Voor Medische Beslissingen Aan Het Levenseinde' in *Palliatieve Zorg En Euthanasie in Belgie: Evaluatie van de Praktijk En de Wetten* (ASP, 2011) 31	Book chapter	Dutch	Non-empirical	Examines the Belgian legal framework for medical decisions at the end of life	Law, professional standards, system design
98	Gastmans, C, 'Caring for a Dignified End of Life in a Christian Health-Care 15 Institution the View of Caritas Catholica Vlaanderen' (2002) 9 *Ethical Perspectives* 134	Article	English	Non-empirical*	Sketches the context in which Caritas Vlaanderen developed their position paper on Care for a Dignified End of Life and reproduces the complete text of the advice	Law, policy
99	Goffin, T, 'Het oordeel van de kinderpsychiater of psycholoog als extra voorwaarde voor het toepassen van euthanasie bij een minderjarige' ['The Opinion of the Child Psychiatrist or Psychologist as Extra Condition for Performing Euthanasia on a Minor'] (2016) 1 *Tijdschrift voor Gezondheidsrecht* 34, 36–37	Article	Dutch	Non-empirical	Critically analyses the constitutional court's 2015 decision on the 2014 amendment to the Belgian Act	Law, professional standards, system design
100	Jans, Jan, 'The Belgian "Act on Euthanasia": Clarifying Context, Legislation, and Practice from an Ethical Point of View' (2005) 25(2) *Journal of the Society of Christian Ethics* 163	Article	English	Non-empirical	Critically compares the Belgian and Dutch euthanasia legislation	Law, policy, training
101	Leleu, YH and G Genicot, 'L'euthanasie En Belgique et Pays-Pas' [2004] (57) *Revue Trimestrielle Droits d'Homme* 10	Article	French	Non-empirical	Presents a comparative analysis of the Belgian and Dutch legislation	Law, system design
102	Lemiengre, Joke et al, 'Impact of Written Ethics Policy on Euthanasia from the Perspective of Physicians and Nurses: A Multiple Case Study in Hospitals' (2010) 1(2) *AJOB Primary Research* 49	Article	English	Empirical	Explores the impact of a written ethics policy on euthanasia on the euthanasia care processes employed by physicians and nurses	Law, policy
103	Lemmens, Trudo, 'The Conflict Between Open-Ended Access to Physician-Assisted Dying and The Protection of the Vulnerable: Lessons from Belgium's Euthanasia Regime in the Post-Carter Era' in Catherine Regia, Laura Khoury and Robert Kouri (eds), *Les Grants Conflits En Droit de La Sante* (Yvon Blais, 2016) 261	Book chapter	English	Non-empirical	Argues that the Canadian federal parliament should not introduce an assisted dying regime such as that in Belgium. Discusses key features of the Belgian system	Law, training, system design
104	Lemmens, Trudo, & Elizabeth Kurz, "The Future of Medically Hastened Death in Canada: Why and How We Should Avoid the Mortal Flaws of Belgium's Euthanasia Regime" in Mark Henaghan & Jesse Wall, Law, Ethics and Medicine: Essays in Honour of Peter Skegg (Wellington: Thompson Reuters, 2016) 96-122	Book chapter	English	Non-empirical	Generates lessons for Canada from a regulatory investigation of Belgian assisted dying practice	Law, system design
105	Meek, C, 'Pharmacy Involvement Where Assisted Suicide and Euthanasia Are Permitted' (2006) 211 *The Pharmaceutical Journal* 614	Article	English	Non-empirical	Investigates pharmacists' involvement in physician assisted dying and assisted dying in countries where one or both are legal, and the potential implications for pharmacy in Britain if assisted suicide were to be legalised	Law, professional standards, system design
106	Vansweevelt, Thierry, 'De euthanasiewet: De ultieme bevestiging van het zelfbeschikkingsrecht of een	Article	Dutch	Non-empirical	Detailed non-empirical investigation of the Belgian assisted dying legal framework	Law, policy, professional

	gecontroleerde keuzevrijheid?’ (2003) 4(22) *Tijdschrift voor Gezondheidsrecht* 63					standards, system design
107	Veny, Ludo M and P Goes, ‘Een wereldprimeur: de uitbreiding van de Euthanasiewet naar niet-ontvoogde minderjarigen’ (2014) 5 *Rechtskundig Weekblad* 163	Article	Dutch	Non-empirical	Critiques and identifies the implications of the 2014 amendment of the Belgian law to minors	Law

* These records extract a source of regulation (e.g. a policy) within the text of the record. The text of the extracted regulatory source was not analysed in this scoping review.
